# Incidence, Clinical Risk Factors, and Pregnancy Outcomes of Trophectoderm‐ and Inner Cell Mass–Poor‐Quality Blastocysts in Single Blastocyst Transfer Cycles: A Retrospective Cohort Study

**DOI:** 10.1002/rmb2.70006

**Published:** 2025-12-30

**Authors:** Longbin Chen, Yangqin Peng, Shuoping Zhang, Yifan Gu, Fei Gong, Ge Lin, Lizhi Leng

**Affiliations:** ^1^ Department of Reproductive and Stem Cell Engineering, School of Basic Medical Central South University Changsha Hunan People's Republic of China; ^2^ Clinical Research Center for Reproduction and Genetics in Hunan Province Reproductive and Genetic Hospital of CITIC‐Xiangya Changsha Hunan People's Republic of China

**Keywords:** early miscarriage rate, intrauterine development, live birth rate, low‐grade blastocysts, risk factors, sex ratio

## Abstract

**Purpose:**

To study incidence, clinical risk factors, and pregnancy outcomes of trophectoderm‐ and inner cell mass–poor‐quality blastocysts in single blastocyst transfer cycles.

**Design:**

A retrospective cohort study included patients who underwent their first single blastocyst transfer cycle. A multivariate log‐binomial regression model with COPY methods was performed.

**Results:**

The incidence of having no transferable high‐quality blastocysts was 33.8% (15 685/46 433). Maternal age, BMI, infertility duration, cycle number, embryo developmental stage, and transfer strategies are independent risk factors that affect the quality of blastocysts (*p* < 0.05). After adjusting for 11 confounding factors, the CPR and LBR significantly decreased across the groups in the order of high‐grade blastocysts, low TE grade, and low ICM grade. Late‐term miscarriage, preterm birth, singleton live birth, low birth weight of singleton, and birth weight of singleton were not statistically different between three groups. Low TE grade blastocysts exhibited significantly higher rates of small gestational sacs compared to high‐grade blastocysts, while low ICM grade blastocysts were associated with a significantly higher incidence of small yolk sacs compared to both high‐grade blastocysts and those with low TE grade.

**Conclusions:**

Differential impacts of blastocyst component quality on early embryonic development.

## 1 Introduction

Routine assisted reproductive techniques include oocyte retrieval, sperm acquisition, fertilization, embryo culture, embryo selection and transfer. Embryo scoring and selection are final critical steps, involving selecting the optimal embryo from multiple embryos for transfer. Blastocyst culture is increasingly used because extended in vitro culture can provide embryologists with more information for embryo selection, and data have shown that single blastocyst transfers have higher pregnancy success rates and lower twin pregnancy rates compared to cleavage‐stage embryo transfers [1]. A common method of embryo scoring is the Gardner Blastocyst Morphology Score [2] in which embryos are scored in combinations based on the degree of expansion of the blastocyst (stage 6 > 5 > 4 > 3), the area and tightness of the inner cell mass (A > B > C), and the number of cells and looseness of the trophectoderm (A > B > C), so that the highest‐scoring embryos can be selected for transfer. It is often assumed that blastocysts graded as 4BB and above are high‐grade blastocysts. However, it has also been suggested that low‐grade blastocysts also have some clinical utility, especially when no high‐grade blastocysts are available [2, 3]. However, current research has primarily focused on high‐quality blastocysts, and our understanding of low‐grade blastocysts remains limited. For instance, it is still unclear how often low‐grade blastocyst transfer becomes necessary in the absence of high‐quality embryos, and which patient characteristics or clinical treatment factors contribute to poor blastocyst quality. Data on pregnancy outcomes and perinatal outcomes for different grades of low‐grade blastocysts are also limited, as most studies group all low‐scoring blastocysts together and compare them with the best blastocysts [4]. In fact, low‐grade blastocysts can be subdivided into three categories: low grade of inner cell mass (ICM = C, TE = A/B), low grade of trophoblast (ICM = A/B, TE = C) and mean difference (ICM = C, TE = C). Among them, CC grade blastocysts are of the worst quality, and the priority of embryo utilization is at the bottom, but the selection of embryos with poor inner cell mass (ICM graded as C) or poor trophectoderm cells (TE graded as C) becomes a controversial topic.

Some scholars argue that after blastocyst implantation into the endometrium, trophectoderm cells are the first to interact with the endometrium, facilitating molecular communication and immune recognition, thereby establishing the maternal‐fetal interface [5, 6, 7]. In contrast, other scholars emphasize that the inner cell mass develops into the fetus, whereas the trophectoderm forms the placenta, which is expelled during labor and delivery [8, 9]. Thus, the inner cell mass grade is considered more critical for successful pregnancy outcomes. The relative importance of morphological scoring for the inner cell mass versus the trophectoderm remains unresolved. Discrepancies in statistical results across studies [10, 11] may arise from variations in treatment protocols (e.g., frozen versus fresh embryo transfer), developmental stages, and blastocyst expansion degrees among the included samples.

Therefore, our study included single blastocyst transfer data from the first transfer cycle to compare the incidence, clinical determinants, and pregnancy outcomes of trophectoderm‐ vs. inner cell mass–poor‐quality blastocysts, and to provide a more accurate and effective transfer strategy for non‐high‐grade blastocyst cycles.

## 2 Materials and Methods

### 2.1 Study Design and Patients

This retrospective study analyzed data from patients who underwent their first single blastocyst transfer cycle at CITIC Xiangya Reproductive and Genetic Hospital between January 1, 2014, and December 31, 2022. Exclusion criteria included the use of donor sperm, donor oocytes, frozen eggs, frozen sperm, and cases with lost follow‐up on pregnancy outcomes. This study was approved by the Institutional Review Board of Reproductive and Genetic Hospital of CITIC‐Xiangya (LL‐SC‐2020‐019).

### 2.2 Stimulation Protocol and Embryo Culture

The ovarian stimulation protocols were performed as described by Tan et al. [12]. When two‐thirds of the follicles reached 18 mm on ultrasonography, the patient was injected with hCG (5000–10 000 IU Pregnyl, Merck, Rahway, NJ, USA) to stimulate the ovaries. Cumulus oocyte complexes (COCs) were collected 34–36 h after hCG administration by transvaginal ultrasound. Fertilization with conventional in vitro fertilization (IVF) or intracytoplasmic sperm injection (ICSI) was chosen based on the quality of the male partner's semen. Fertilization was assessed at 16–18 h after insemination. Zygotes with two pronuclei (2PN) were identified as normally fertilized and transferred to a cleavage medium (G1.5, Vitrolife). On Day 3, embryos were scored using Puissant's criterion [13]. Embryos were transferred, cryopreserved, or cultured to the blastocyst stage according to clinical indications and the willingness of the patient.

### 2.3 Blastocyst Culture and Scoring

During cycles of blastocyst culture, embryos were transferred to be placed in G2 culture medium (Vitrolife, Sweden) for 2–3 days. Blastocysts were graded according to the Gardner and Schoolcraft system [14] and consensus [15]. Groups were defined as follows: Group 0 (High) = AA/AB/BA/BB; Group 1 (Low‐TE) = AC/BC; Group 2 (Low‐ICM) = CA/CB. CC embryos were ineligible for transfer and were excluded. Blastocysts with a score ≥ 4BC or 4CB on Day 5/6 were considered suitable for transfer, and the remaining blastocysts (≥ 4BC or 4CB) on Days 5 to 7 were cryopreserved. Morphology was recorded pre‐vitrification. For biopsied‐blast, the TE biopsy was performed as described previously [12]. Blastocysts were vitrified within 1–2 h after TE biopsy, and biopsied TE cells were submitted for next generation sequencing.

### 2.4 Embryo Vitrification, Thawing, and Transfer

The expanded, hatching, and completely hatched blastocysts were artificially collapsed before vitrification using Zilos TK laser, while the cleavage‐stage embryos were not required. Embryos were vitrified and thawed using the Kitazato vitrification kit (Kitazato Biopharma, Shizuoka, Japan) in combination with closed High Security Vitrification Straws (Cryo Bio System, France). For blast‐thawed‐D3, all thawed cleavage‐stage embryos were transferred to blastocyst medium (G2.5, Vitrolife). Blastocysts scored ≥ 4bc or 4CB on Days 5 and 6 were considered suitable for transfer. For thawed‐blast and biopsied‐blast, all embryos were thawed on the day of transfer. Blastocysts were not graded after thawing, and the pre‐vitrification scores were adopted in this study. The thawed blastocysts were transferred to G2.5 medium and cultured for 2–6 h. The re‐expanded blastocysts were considered as survival and suitable for transfer.

When both A/BC and CA/B were available at the first transfer, A/BC blastocyst transplantation is given priority clinically. Only when A/BC is aneuploid and CA/B is euploid blastocyst, or A/BC cryo resuscitation fails and CA/B is successful, CA/B is selected for transplantation. We report the counts of only‐A/BC, only‐CA/B, and both‐available at the index transfer (Table S4), and conduct sensitivity analyses restricted to the both‐available subset as well as propensity score–weighted analyses of the clinical choice (CA/B vs. A/BC).

### 2.5 Ultrasonic Inspection

We included ultrasound data from patients 6 weeks after conception; only data from patients with early miscarriages were included in the analysis. The size of the gestational sac is calculated based on the average of three measurements of its vertical diameter taken with a caliper placed on the inner edge of the trophoblast. The size of the yolk sac is calculated based on the average of three measurements of its vertical diameter relative to the center of the yolk sac wall. The length of the embryonic pole is measured along the anterior–posterior axis. The embryonic heart rate is measured using an electronic caliper from frozen M‐mode images. The ultrasound parameters were compared with the biometric reference data provided in the report by Papaioannou et al., which provided reference values for embryo length, embryo heart rate, gestational sac diameter, and yolk sac diameter relative to gestational age (GA) for normal pregnancies [16]. GS means gestational sac, CRL means crown‐rump length, EHR means embryonic heart rate. Small GS is defined as ≤ 26 mm 6 weeks after embryo transfer. Short CRL is defined as having a germ/crown‐rump length ≤ 14 mm 6 weeks after embryo transfer. Low EHR is defined as ≤ 120 bpm 6 weeks after embryo transfer. Large or small YS is defined as > 6 mm or < 3 mm 6 weeks after embryo transfer.

### 2.6 Outcome Measures

All clinical outcomes were defined according to the reported guidelines [17]. The primary outcome was the LBR (defined as the presence of respiration or any other signs of life, such as breathing, heartbeat, random muscle movements, after the foetus has been completely expelled from the woman's body or removed and separated from her body after 20 weeks of gestational age). Other outcomes included the rates of biochemical pregnancy (BPR, defined as pregnancy loss occurring within 5 weeks of gestation), clinical pregnancy (CPR, defined as a visible heartbeat by ultrasound), early‐term miscarriage (EMR, defined as the spontaneous loss of an intra‐uterine pregnancy ≤ 12 weeks of gestational age), late‐term miscarriage (LMR, defined as termination of pregnancy between 12 and 28 weeks of gestation), and obstetric outcomes, including the rates of preterm birth (defined as delivery between 28 weeks and 37 weeks of gestation), singleton live birth, sex ratio of singleton live birth (defined as males divided by females), low birthweight rate of singleton live birth (defined as weighing less than 2500 g in the first 24 h after birth), and birthweight of singleton live birth (defined as weight in the first 24 h of birth).

### 2.7 Statistical Analyses

Continuous variables are described using median (interquartile range), and categorical variables are described using frequencies and percentages (*n* (%)). Kruskal–Wallis rank sum test, Pearson's Chi‐squared test, or Fisher's exact test were used to compare baseline characteristics, pregnancy and obstetric outcomes between the three groups of different grade combinations of inner cell mass (ICM) and trophectoderm (TE) for the study population.

We used the “NNET” package of R software to conduct multivariable multinomial logistic regression to identify independent predictors of embryonic grade (group 0 as reference group, group 1 and group 2) and maternal age, body mass index (BMI), infertility duration, number of treatment cycles, antral follicle count (AFC), anti‐mullerian hormone (AMH), infertility diagnosis (divided into tubal disorders, ovulatory disorders, endometrial factors, male factors, more than one cause of infertility, and unexplained infertility), the type of single blastocyst transfer cycles (divided into fresh blastocyst cycle, biopsied blastocyst cycle, thawed blastocyst cycle, and blastocyst from thawed cleavage embryo cycle), endometrial thickness on the day before transplantation (EM), blastocyst developmental stage (Day 5 and 6), and degree of blastocyst expansion (grade 3, 4, 5, and 6) were considered to be potential predictors; odds ratios (ORs) and 95% confidence intervals (CIs) were calculated.

We used the “PROC GENMOD” program of SAS software version 9.4 to perform a multivariate log‐binomial regression model with COPY method [18, 19] to estimate standardized risk ratios (RR) with robust variance of three groups for reproductive and neonatal dichotomous outcomes and used the “glm” package of R software version 4.3.2 to perform a multivariate linear regression model for continuous outcomes after adjusting for above‐mentioned potential confounders, including maternal age, BMI, infertility duration, number of treatment cycles, AFC, AMH, infertility diagnosis and so on.

We further used the “propensity scores (PS) weight” package of R software for sensitivity analysis to perform five propensity score weighting methods [20] namely inverse probability of treatment weights (IPW), treated weights (Treated), overlap weights (OW), matching weights (MW), and entropy weights (EW), based on the propensity scores (PS) estimation of participants to balance the differences in baseline characteristics between the three groups (group 0, 1, 2). IPW, whose target population is the combined treatment and control group represented by the observed sample, and the target estimand is the average treatment effect among the combined population (ATE). Treated, whose target population is the treated group and target estimand is the average treatment effect for the treated population (ATT). Treated weights can be viewed as a special case of IPW because it inversely weights the control group. OW, whose target population is the subpopulation with the most overlap in the observed covariates between treatment and control groups. In medicine, this is known as the population in clinical equipoise and is the population eligible to be enrolled in randomized clinical trials. The target estimand of OW is the average treatment effect for the overlap population (ATO). MW and EW focus on target populations with substantial overlap between treatment groups. Though having similar operating characteristics, MW and EW do not possess the same theoretical optimality as OW and are less used in practice [21]. We used aforementioned potential confounders to calculate PS for participants and used density plots of generalized PS to display the estimated probability to receive each treatment level. Baseline characteristics were compared between the three groups for the unweighted population and the weighted target population, respectively. Between‐group equilibrium of covariates was assessed using standardized mean difference (SMD) and visualized via Love plot based on the absolute standardized difference (ASD) or the target population standardized difference (PSD) metric, with a threshold of < 0.1 for these three indicators considering to be better between‐group equilibrium [22] (see Love plots in Figure S4, PS overlap plots in Figure S5, ESS in Table S7, Maximum weights in Table S8, the trimming thresholds = 0.067). Causal relative risk (RR) or weighted difference‐in‐means (DIF) with 95% confidence intervals (CIs) for all pairwise comparisons of average potential dichotomous outcomes on the log scale or of average potential continuous outcomes were estimated respectively and visualized via forestplot.

In addition, we conduct optimal propensity score trimming [22, 23] to address the extreme weights problem through excluding units with estimated (generalized) propensity scores close to zero (or one) for another sensitivity analysis. We estimated the causal effects based on the data with trimming, and the analysis with the trimmed data followed the exact same steps as described previously. All statistical significance levels were set at two‐sided *p* < 0.05.

## 3 Results

**FIGURE 1:**
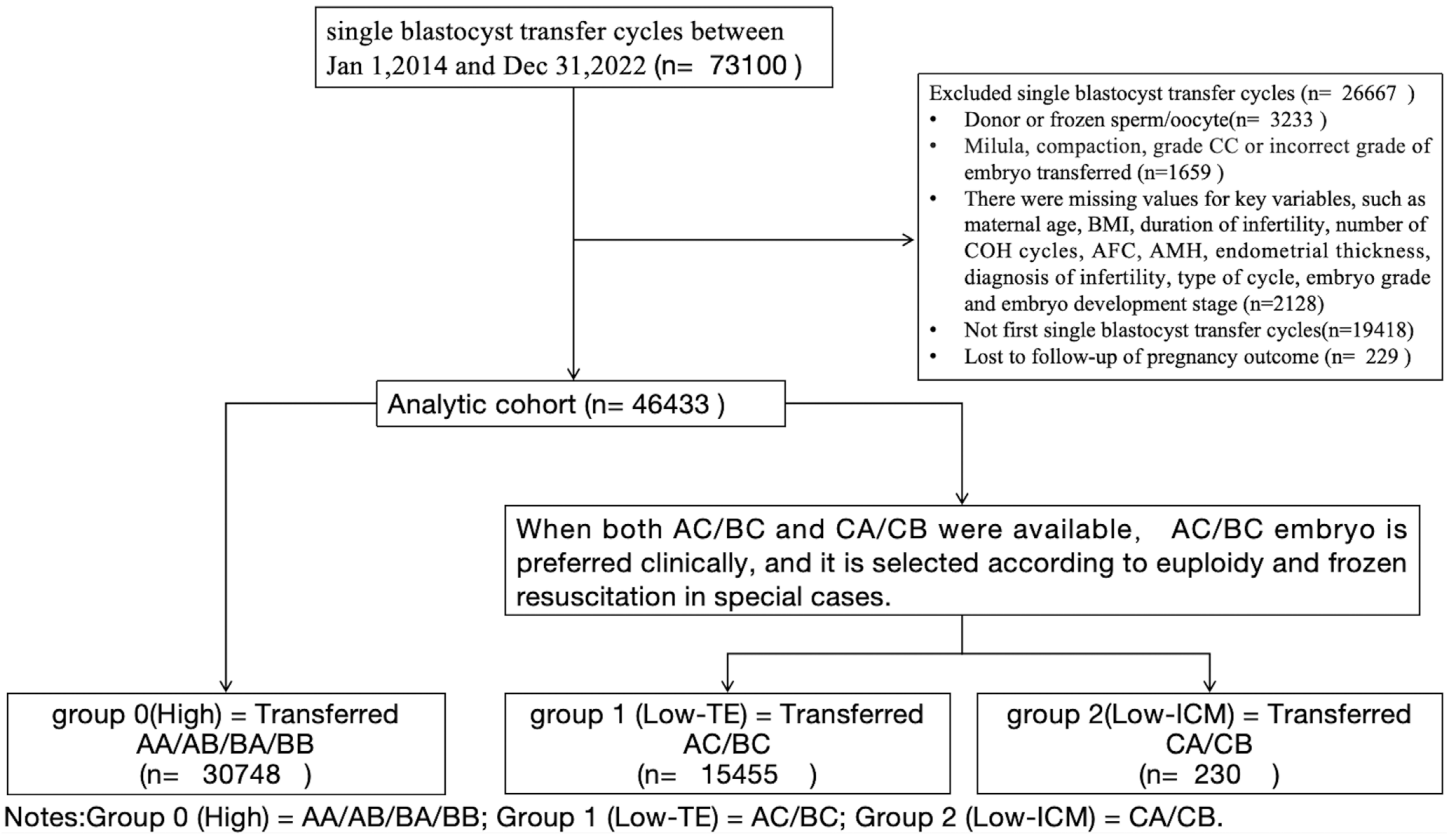
Frame diagram of grouping.

We included a total of 46 433 patients who underwent a single blastocyst transfer—either frozen or fresh—in their first embryo transfer cycle. Among them, 30 748 patients were in Group 0. The incidence of having no high‐quality blastocysts was 33.8% (15 685/46 433), which means 33.8% cycles where blastocysts were obtained but all were low quality. Of these, 15 455 patients were classified into Group 1, accounting for 98.5%, and 230 patients were in Group 2, accounting for 1.5% (see Figure 1). Demographic and treatment characteristics are presented in Table 1, which showed except for endometrial thickness (EM), others like maternal age (age at egg collection), BMI, duration of infertility, number of ovulation induction cycles, AFC, AMH, cause of infertility, embryo development stage, embryo expansion degree and type of transferred cycle were significantly different between three groups. All of five propensity score weighting process resulted in a good balance for all covariates (SMD < 0.1; see Table S1).

**TABLE 1:** Patient demographic and treatment characteristics and neonatal outcomes by 3 embryo types of single blastocyst transfer cycles.

	ALL blastocysts, *N* = 46 433	Group 0, *N* = 30 748	Group 1, *N* = 15 455	Group 2, *N* = 230	SMD	p. Overall	p. Trend	p. Group 1 vs. 0	p. Group 2 vs. 0	p. Group 2 vs. 1
Maternal age (y)	32.0 [29.0; 35.0]	32.0 [29.0; 35.0]	32.0 [29.0; 36.0]	33.0 [30.0; 37.0]	0.25	< 0.001	< 0.001	< 0.001	0.001	0.075
Maternal body mass index (kg/m^2^)	21.6 [20.0; 23.3]	21.6 [20.0; 23.3]	21.7 [20.0; 23.4]	22.0 [20.4; 23.4]	0.04	0.009	0.002	0.010	0.446	0.542
Infertility duration (years)	3.00 [2.00; 5.00]	3.00 [2.00; 5.00]	3.00 [2.00; 5.00]	3.00 [1.00; 5.00]	0.13	< 0.001	< 0.001	< 0.001	0.413	0.026
Treatment cycles	1.00 [1.00; 1.00]	1.00 [1.00; 1.00]	1.00 [1.00; 1.00]	1.00 [1.00; 2.00]	0.29	< 0.001	< 0.001	< 0.001	< 0.001	0.014
Antral follicle counts	20.0 [12.0; 30.0]	21.0 [13.0; 30.0]	17.0 [10.0; 29.0]	16.5 [8.00; 29.0]	0.33	< 0.001	< 0.001	< 0.001	< 0.001	0.063
Antimullerian hormone (ng/mL)	4.17 [2.34; 7.06]	4.44 [2.57; 7.38]	3.62 [1.96; 6.34]	2.80 [1.47; 4.30]	0.53	< 0.001	< 0.001	< 0.001	< 0.001	< 0.001
Endometrial thickness (mm)	11.7 [10.5; 13.1]	11.8 [10.5; 13.1]	11.7 [10.5; 13.0]	11.7 [10.5; 12.7]	0.08	0.348	0.206	0.441	0.441	0.441
Infertility diagnosis						< 0.001	< 0.001	< 0.001	0.105	< 0.001
Endometrial factors	2770/46 433 (5.97%)	1805/30 748 (5.87%)	942/15 455 (6.10%)	23/230 (10.0%)	Reference					
Unexplained	4726/46 433 (10.2%)	3537/30 748 (11.5%)	1160/15 455 (7.51%)	29/230 (12.6%)	0.17					
More than one causes	12 687/46 433 (27.3%)	8109/30 748 (26.4%)	4517/15 455 (29.2%)	61/230 (26.5%)	0.06					
Male factor	4032/46 433 (8.68%)	2893 (9.41%)	1115 (7.21%)	24/230 (10.4%)	0.11					
Ovulatory disorders	1582/46 433 (3.41%)	1240/30 748 (4.03%)	334/15 455 (2.16%)	8/230 (3.48%)	0.11					
Tubal disorders	20 636/46 433 (44.4%)	13164/30 748 (42.8%)	7387/15 455 (47.8%)	85/230 (37.0%)	0.22					
Developmental stage						< 0.001	0.000	< 0.001	< 0.001	< 0.001
D5	19 379/46 433 (41.7%)	13 680/30 748 (44.5%)	5673/15 455 (36.7%)	26/230 (11.3%)	Reference					
D6	27 054/46 433 (58.3%)	17 068/30 748 (55.5%)	9782/15 455 (63.3%)	204/230 (88.7%)	0.75					
Expansion							0.000	0.000	0.001	< 0.001
3	150/46 433 (0.32%)	44/30 748 (0.14%)	106/15 455 (0.69%)	0/230 (0.00%)	Reference					
4	32 165/46 433 (69.3%)	19681/30 748 (64.0%)	12 362/15 455 (80.0%)	122/230 (53.0%)	0.58					
5	5527/46 433 (11.9%)	3923/30 748 (12.8%)	1577/15 455 (10.2%)	27/230 (11.7%)	0.08					
6	8591/46 433 (18.5%)	7100/30 748 (23.1%)	1410/15 455 (9.12%)	81/230 (35.2%)	0.65					
Group						0.000	0.000	0.000	< 0.001	< 0.001
Fresh	9398/46 433 (20.2%)	6355/30 748 (20.7%)	3028/15 455 (19.6%)	15/230 (6.52%)	Reference					
PGT	12 709/46 433 (27.4%)	10 312/30 748 (33.5%)	2321/15455 (15.0%)	76/230 (33.0%)	0.42					
Thawed blastocysts	16 440/46 433 (35.4%)	8835/30 748 (28.7%)	7490/15 455 (48.5%)	115/230 (50.0%)	0.44					
Thawed cleavage	7886/46 433 (17.0%)	5246/30 748 (17.1%)	2616/15 455 (16.9%)	24/230 (10.4%)	0.19					
Clinical pregnancy						< 0.001	0.000	< 0.001	< 0.001	< 0.001
Yes	17 761/46 433 (38.3%)	10 076/30 748 (32.8%)	7516/15 455 (48.6%)	169/230 (73.5%)						
No	28 672/46 433 (61.7%)	20 672/30 748 (67.2%)	7939/15 455 (51.4%)	61/230 (26.5%)						
Live birth						< 0.001	0.000	< 0.001	< 0.001	< 0.001
Yes	22 935/46 433 (49.4%)	13 494/30 748 (43.9%)	9252/15 455 (59.9%)	189/230 (82.2%)						
No	23 498/46 433 (50.6%)	17 254/30 748 (56.1%)	6203/15 455 (40.1%)	41/230 (17.8%)						

	ALL blastocysts, *N* = 28 672	Group 0, *N* = 20 672	Group 1, *N* = 7939	Group 2, *N* = 61	p. Overall	p. Trend	p. 0 vs. 1	p. 0 vs. 2	p. 1 vs. 2
Ectopic pregnancy					0.744	0.477	1.000	1.000	1.000
Yes	28 456/28 672 (99.2%)	20512/20 672 (99.2%)	7883/7939 (99.3%)	61/61 (100%)					
No	216/28 672 (0.75%)	160/20672 (0.77%)	56/7939 (0.71%)	0/61 (0.00%)					
Early miscarriage					< 0.001	0.000	< 0.001	< 0.001	0.026
Yes	24 726/28 672 (86.2%)	18 152/20 672 (87.8%)	6531/7939 (82.3%)	43/61 (70.5%)					
No	3946/28 672 (13.8%)	2520/20672 (12.2%)	1408/7939 (17.7%)	18/61 (29.5%)					
Late miscarriage					0.812	0.548	1.000	1.000	1.000
Yes	27 660/28 672 (96.5%)	19 934/20 672 (96.4%)	7667/7939 (96.6%)	59/61 (96.7%)					
No	1012/28 672 (3.53%)	738/20 672 (3.57%)	272/7939 (3.43%)	2/61 (3.28%)					

	ALL blastocysts, *N* = 23 498	Group 0, *N* = 17 254	Group 1, *N* = 6203	Group 2, *N* = 41	p. Overall	p. Trend	p. 0 vs. 1	p. 0 vs. 2	p. 1 vs. 2
Preterm birth					0.451	0.809	0.981	0.406	0.406
Yes	21 325/23 498 (90.8%)	15 661/17 254 (90.8%)	5629/6203 (90.7%)	35/41 (85.4%)					
No	2173/23498 (9.25%)	1593/17254 (9.23%)	574/6203 (9.25%)	6/41 (14.6%)					
Normal delivery					0.451	0.809	0.981	0.406	0.406
Yes	2173/23 498 (9.25%)	1593/17 254 (9.23%)	574/6203 (9.25%)	6/41 (14.6%)					
No	21 325/23 498 (90.8%)	15 661/17 254 (90.8%)	5629/6203 (90.7%)	35/41 (85.4%)					
Plurality (singleton/twin)					0.364	0.360	0.499	0.499	0.499
1	23 117/23 498 (98.4%)	16 966/17 254 (98.3%)	6111/6203 (98.5%)	40/41 (97.6%)					
2	381/23 498 (1.62%)	288/17 254 (1.67%)	92/6203 (1.48%)	1/41 (2.44%)					

	ALL blastocysts, *N* = 23 117	Group 0, *N* = 16 966	Group 1, *N* = 6111	Group 2, *N* = 40	p. Overall	p. Trend	p. 0 vs. 1	p. 0 vs. 2	p. 1 vs. 2
Male sex among singleton live births					< 0.001	0.000	< 0.001	0.714	0.714
0	9414/23 117 (40.7%)	6519/16 969 (38.4%)	2878/6111 (47.1%)	17/40 (42.5%)					
1	13 703/23 117 (59.3%)	10 447/16 969 (61.6%)	3233/6111 (52.9%)	23/40 (57.5%)					
Birth weight of singleton live births (g)	3300 [3000; 3600]	3300 [3000; 3600]	3300 [3000; 3600]	3400 [3138; 3850]	0.021	0.032	0.065	0.126	0.126
Birth weight grouping of singleton live births						0.937	0.701	0.701	0.701
LBW (< 2500 g)	1049 (4.54%)	764 (4.50%)	283 (4.63%)	2 (5.00%)					
Macrosomia (≥ 4000 g)	1937 (8.38%)	1437 (8.47%)	496 (8.12%)	4 (10.0%)					
Normal	20131 (87.1%)	14765 (87.0%)	5332 (87.3%)	34 (85.0%)					

*Note:* Group 0 (High) = AA/AB/BA/BB; Group 1 (Low‐TE) = AC/BC; Group 2 (Low‐ICM) = CA/CB. Birth weight of singleton live births (g) measured within 24 h.

### 3.1 Patient and Clinical Factors Associated With Poor Blastocyst Quality

Multivariate logistic regression models were employed to identify risk factors associated with blastocyst quality (see Table 2). Compared to group 0, several factors were significantly associated with an increased likelihood of producing blastocysts with low trophectoderm (TE) grades. Specifically, advanced maternal age (OR = 1.104, 95% CI: 1.079–1.130, *p* < 0.001), higher BMI (OR = 1.029, 95% CI: 1.008–1.051, *p* = 0.006), longer duration of infertility (OR = 1.035, 95% CI: 1.014–1.056, *p* = 0.001), greater number of treatment cycles (OR = 1.066, 95% CI: 1.045–1.089, *p* < 0.001), and prolonged embryonic development time (OR = 2.495, 95% CI: 2.331–2.670, *p* < 0.001) were all significantly associated with an increased risk of group 1. Additionally, unexplained infertility was identified as an independent risk factor (OR = 1.102, 95% CI: 1.015–1.197, *p* = 0.021).

**TABLE 2:** Predictors of Group 1 (Low‐TE) and Group 2 (Low‐ICM) vs Group 0 (High): Multivariable multinomial logistic regression (RRR, 95% CI).

Predictors	Group 1 vs 0: RRR (95% CI)	*p*	Group 2 vs 0: RRR (95% CI)	*p*
Age	1.104 (1.079, 1.13)	0	1.076 (0.933, 1.241)	0.312
BMI	1.029 (1.008, 1.051)	0.006	1.012 (0.883, 1.159)	0.863
Infertility duration	1.035 (1.014, 1.056)	0.001	0.921 (0.804, 1.057)	0.242
Cycle number	1.066 (1.045, 1.089)	0	1.129 (1.035, 1.232)	0.007
AFC	0.846 (0.821, 0.871)	0	1.161 (0.949, 1.422)	0.147
AMH	0.977 (0.95, 1.004)	0.096	0.476 (0.36, 0.63)	0
*Tubal disorders* ^*a*^				
Ovulatory disorders	0.682 (0.598, 0.778)	0	1.523 (0.709, 3.274)	0.281
Endometrial factors	0.871 (0.798, 0.952)	0.002	1.588 (0.995, 2.534)	0.052
Male factors	0.993 (0.916, 1.076)	0.862	1.446 (0.9, 2.322)	0.127
Unexplained infertility	1.102 (1.015 1.197)	0.021	1.326 (0.836, 2.104)	0.231
More than one causes	0.974 (0.928, 1.023)	0.291	1.243 (0.891, 1.733)	0.2
*Embryo development stage = D5* ^*a*^				
Embryo development stage = D6	2.495 (2.331, 2.67)	0	8.47 (4.317, 16.617)	0
*Embryo expansion 3* ^*a*^				
Embryo expansion 4	0.2 (0.14, 0.287)	0	7264666852.684 (5748635029.635, 9180507060.967)	0
Embryo expansion 5	0.177 (0.123, 0.255)	0	9483800659.792 (6721386853.124, 13381535227.787)	0
Embryo expansion 6	0.086 (0.06, 0.124)	0	14653498760.397 (10970596448.568, 19572775913.109)	0
*Fresh* ^*a*^				
PGT cycle	0.279 (0.25, 0.31)	0	0.169 (0.068, 0.421)	0
Thawed blastocyst cycle	0.775 (0.715, 0.839)	0	0.57 (0.245, 1.323)	0.191
Thawed cleavage embryo cycle	0.808 (0.755, 0.865)	0	0.718 (0.325, 1.584)	0.411

^a^ Reference categories. Group 0 (High) = AA/AB/BA/BB; Group 1 (Low‐TE) = AC/BC; Group 2 (Low‐ICM) = CA/CB.

Conversely, a higher antral follicle count (AFC) (OR = 0.846, 95% CI: 0.821–0.871, *p* < 0.001) and greater degree of blastocyst expansion were significantly associated with a reduced likelihood of low TE grade blastocyst formation (see Table 2 for details). In terms of cycle type, compared to fresh embryo transfer cycles, both preimplantation genetic testing (PGT) cycles (OR = 0.279, 95% CI: 0.250–0.310, *p* < 0.001) and frozen embryo transfer (FET) cycles—including thawed blastocyst cycles (OR = 0.775, 95% CI: 0.715–0.839, *p* < 0.001) and thawed cleavage‐stage embryo cycles (OR = 0.808, 95% CI: 0.755–0.865, *p* < 0.001)—were significantly associated with a lower probability of group 1.

Similarly, when analyzing factors associated with group 2, the number of treatment cycles (OR = 1.129, 95% CI: 1.035–1.232, *p* < 0.001) and longer embryonic development time (OR = 8.470, 95% CI: 4.317–16.617, *p* < 0.001) were significantly associated with increased risk. In contrast, higher anti‐Müllerian hormone (AMH) levels were associated with a significantly reduced risk of group 2 (OR = 0.476, 95% CI: 0.360–0.630, *p* < 0.001). Furthermore, PGT cycles were associated with a lower incidence of group 2 compared to fresh embryo cycles (OR = 0.169, 95% CI: 0.068–0.421, *p* < 0.001).

### 3.2 Pregnancy Outcomes and Perinatal Outcomes With Non‐Trim Data

**FIGURE 2:**
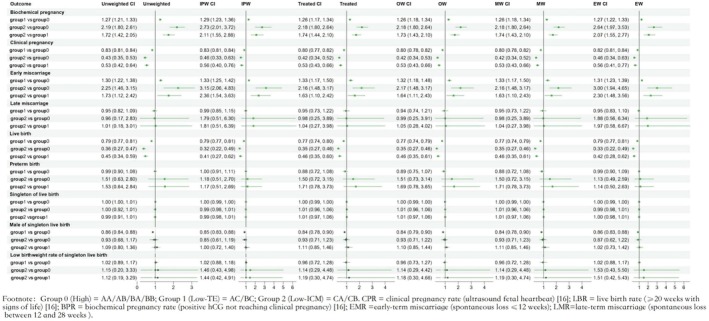
Forest plot of dichotomous outcome comparisons between the three groups.

Estimating standardized risk ratios and robust variance for three groups of reproductive and neonatal categorical outcomes using multivariate log‐binomial regression models were shown in Figure 2. When compared with group 0, group 1 and group 2 had significantly lower LBR and CPR (LBR: group 1 vs. group 0, RR = 0.79 (0.77, 0.81); group 2 vs. group 0, RR = 0.36 (0.27, 0.47); CPR: group 1 vs. group 0, RR = 0.83 (0.81, 0.84); group 2 vs. group 0, RR = 0.43 (0.35, 0.53); *p* < 0.05). When compared with group 1, group 2 had significantly lower LBR and CPR (LBR: group 2 vs. group 1, RR = 0.45 (0.34, 0.59); CPR: group 2 vs. group 1, RR = 0.53 (0.42, 0.64); *p* < 0.05), indicating that the LBR and CPR of the three groups were decreasing according to the order of group 0, group 1 and group 2. When compared with group 0, group 1 and group 2 had significantly higher BPR and EMR (BPR: group 1 vs. group 0, RR = 1.27 (1.21, 1.33); group 2 vs. group 0, RR = 2.19 (1.80, 2.61); EMR: group 1 vs. group 0, RR = 1.30 (1.22, 1.38); group 2 vs. group 0, RR = 2.25 (1.46, 3.15); *p* < 0.05). When compared with group 1, group 2 had significantly higher BPR and EMR (BPR: group 2 vs. group 1, RR = 1.72 (1.42, 2.05); EMR: group 2 vs. group 1, RR = 1.73 (1.12, 2.42); *p* < 0.05). Indicating that the BPR and EMR of the three groups increased sequentially according to the order of group 0, group 1 and the group 2. Other dichotomous outcome indicators including late miscarriage, preterm, singleton live birth and low birth weight were not significantly different among the three groups. It is worth mentioning that the rate of male of single live births was significantly lower in the group of group 1 than in the group of group 0, RR = 0.86 (0.84, 0.88), *p* < 0.05. Comparative analysis of the continuous variable birth weight using multiple linear regression equations after correcting for confounders revealed no significant differences with *p* greater than 0.05 (Figure S1).

Five propensity score weighting methods named IPW, Treated, OW, MW, and EW were used to balance the differences in baseline characteristics among the three groups (see Table S2). The results obtained were consistent with the above, with LBR and CPR decreasing in the order of group 0, group 1, and group 2, while BPR and EMR increasing in the same order, as shown in Table S2. Also, the rate of male single live births was significantly lower in the low TE grade group than in the high‐grade blastocyst.

### 3.3 Pregnancy Outcomes and Perinatal Outcomes With Trimmed Data

We solved the extreme weighting problem by eliminating units with estimated propensity scores close to zero or one, resulting in trimmed data to conduct similar sensitivity analysis following the above methodology. Similar to the non‐trimmed data, the LBR and CPR of the three groups were decreasing according to the order of group 0, group 1 and group 2 (LBR: group 2 vs. group 0, RR = 0.36 (0.26, 0.47), group 2 vs. group 1, RR = 0.46 (0.34, 0.60); CPR: group 2 vs. group 0, RR = 0.42 (0.33, 0.52), group 2 vs. group 1, RR = 0.51 (0.40, 0.63)); the BPR and EMR of the three groups were increasing according to the order of group 0, group 1 and group 2 (BPR: group 2 vs. group 0, RR = 2.12 (1.72, 2.54), group 2 vs. group 1, RR = 1.70 (1.39, 2.05); EMR: group 2 vs. group 0, RR = 2.15 (1.36, 3.07), group 2 vs. group 1, RR = 1.71 (1.08, 2.45)), with *p*‐values less than 0.05 (Figure 2). Other outcomes were not significantly different among the three groups with the exception of the difference in the rates of male single live births between group 0 and group 1 (Figures S2 and S3). The results of sensitivity analyses using the five weightings were also consistent with the above (Table S3).

Clinical selection between low‐TE vs. low‐ICM may drive group imbalance. To test its impact, we compared the pregnancy outcomes and neonatal outcomes between the two groups in the both‐available subset (Table S5) and conducted propensity score–weighted analyses of the two groups (low TE grade vs. low ICM grade) (Table S6). The results of the two tables were similar. There is no selective shift in theory.

The calendar year was included in the confounding factors for adjustment, while taking into account the possible collinearity between Day (5/6) and expansion (3–6), the culture days were removed, and the results are shown in Table S9.

PGT cycles account for 27.4% of the dataset, and PGT inherently introduces selection bias by preferentially biopsying morphologically good embryos and transferring only euploid embryos. To demonstrate the robustness, we compared the pregnancy outcomes and neonatal outcomes between the three groups within PGT cycles only (Table S10), excluding all PGT cycles (Table S11) and compared the low‐grade blastocyst performance between PGT and non‐PGT cycles (Tables S12 and S13). The results showed that the observed differences are independent of PGT‐related biases.

### 3.4 Intrauterine Development During Early Pregnancy

**FIGURE 3:**
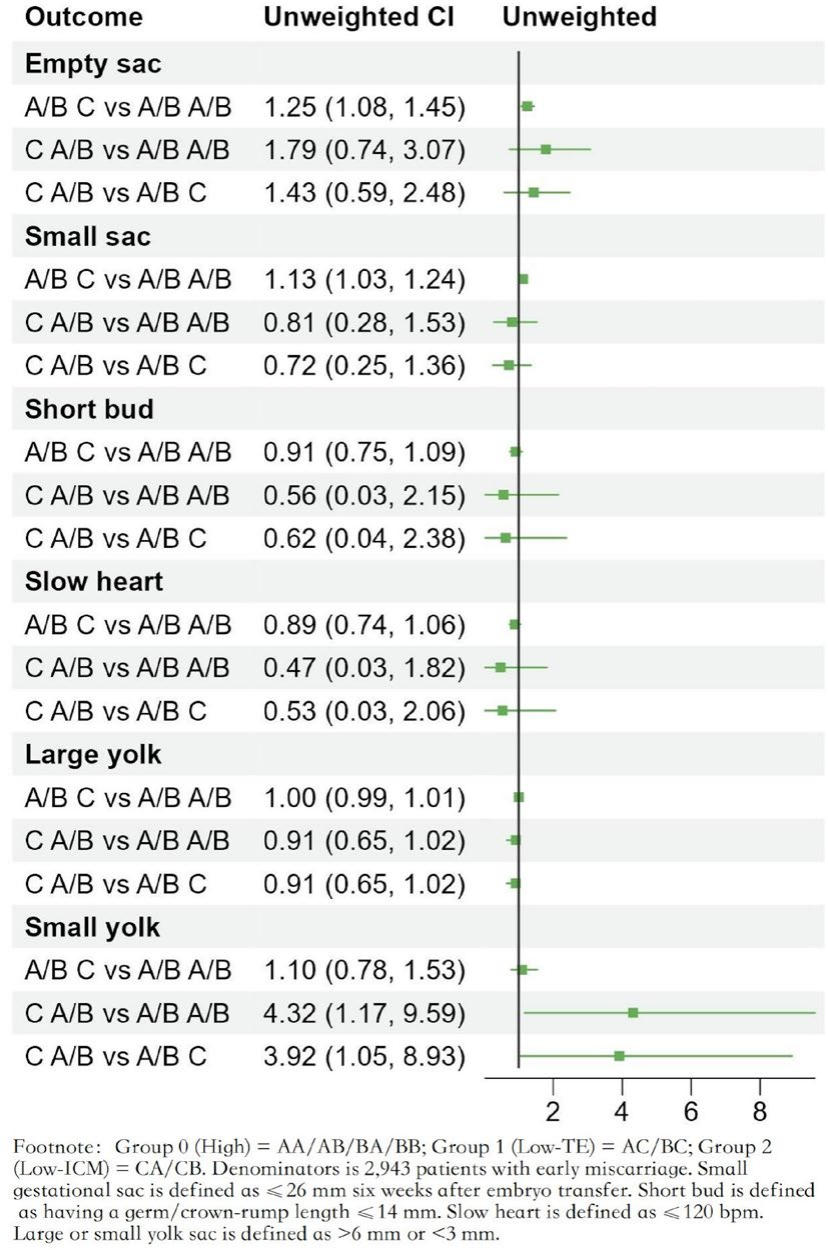
Forest plot for comparison of intrauterine growth indicators between the three groups.

We further analyzed the early pregnancy intrauterine development of embryos with different morphological scores in 2943 patients with early miscarriage. Compared with group 0, group 1 has a significantly higher rate of empty gestational sacs (RR = 1.25 (1.08, 1.45), *p* = 0.003) and small gestational sacs (RR = 1.13 (1.03, 1.24), *p* = 0.013). Compared with group 0, the incidence of small yolk sacs is significantly higher in group 2 (RR = 4.32 (1.17, 9.59), *p* = 0.004). The same trend was observed in comparison with group 1 (RR = 3.92 (1.05, 8.93), *p* = 0.009; Figure 3). Other indicators such as short embryo length, slow fetal heart rate, and large yolk sac showed no significant differences among the three groups (Table 3).

**TABLE 3:** Early ultrasound indicators: Log‐binomial (COPY) risk ratios (RR, 95% CI) with adjusted absolute percentages (95% CI).

Outcome	Unweighted, adjusted RR (95% CI)	*p*	Overlap‐weighted RR (95% CI)	*p*	IPW‐weighted RR (95% CI)	*p*
*Empty GS*						
Group 1 vs. 0	1.25 (1.08, 1.45)	0.003	1.01 (0.96, 1.07)	0.606	1.04 (1.01, 1.07)	0.004
Group 2 vs. 0	1.79 (0.74, 3.07)	0.100	1.13 (0.93, 1.37)	0.209	1.12 (0.90, 1.39)	0.320
Group 1 vs. 2	1.43 (0.59, 2.48)	0.312	1.12 (0.92, 1.36)	0.275	1.07 (0.86, 1.34)	0.529
*Small GS*						
Group 1 vs. 0	1.13 (1.03, 1.24)	0.013	1.01 (0.96, 1.07)	0.691	1.03 (1.00, 1.06)	0.041
Group 2 vs. 0	0.81 (0.28, 1.53)	0.615	0.94 (0.77, 1.14)	0.527	0.94 (0.76, 1.16)	0.565
Group 1 vs. 2	0.72 (0.25, 1.36)	0.429	0.93 (0.76, 1.13)	0.467	0.91 (0.73, 1.13)	0.400
*Short CRL for GA*						
Group 1 vs. 0	0.91 (0.75, 1.09)	0.316	1.00 (0.95, 1.04)	0.830	0.99 (0.96, 1.01)	0.239
Group 2 vs. 0	0.56 (0.03, 2.15)	0.547	0.94 (0.83, 1.07)	0.372	0.89 (0.85, 0.93)	0.000
Group 1 vs. 2	0.62 (0.04, 2.38)	0.617	0.95 (0.83, 1.08)	0.423	0.90 (0.86, 0.95)	0.000
*Low EHR for GA*						
Group 1 vs. 0	0.89 (0.74, 1.06)	0.184	1.01 (0.96, 1.06)	0.594	0.98 (0.96, 1.01)	0.194
Group 2 vs. 0	0.47 (0.03, 1.82)	0.433	0.92 (0.81, 1.05)	0.237	0.87 (0.83, 0.92)	0.000
Group 1 vs. 2	0.53 (0.03, 2.06)	0.511	0.91 (0.80, 1.04)	0.181	0.89 (0.84, 0.93)	0.000
*Enlarged YS*						
group 1 vs. 0	1.00 (0.99, 1.01)	0.375	1.00 (0.98, 1.01)	0.927	1.00 (0.99, 1.01)	0.964
Group 2 vs. 0	0.91 (0.65, 1.02)	0.404	0.95 (0.85, 1.06)	0.326	0.89 (0.74, 1.07)	0.225
Group 1 vs. 2	0.91 (0.65, 1.02)	0.383	0.95 (0.85, 1.06)	0.334	0.89 (0.74, 1.07)	0.225
*Small YS*						
Group 1 vs. 0	1.10 (0.78, 1.53)	0.573	0.97 (0.94, 1.00)	0.059	1.00 (0.99, 1.02)	0.809
Group 2 vs. 0	4.32 (1.17, 9.59)	0.004	1.15 (0.96, 1.39)	0.136	1.18 (0.92, 1.53)	0.197
Group 1 vs. 2	3.92 (1.05, 8.93)	0.009	1.19 (0.99, 1.43)	0.070	1.18 (0.91, 1.52)	0.203

*Note:* Unweighted = log‐binomial COPY (no PS weights). Group 0 (High) = AA/AB/BA/BB; Group 1 (Low‐TE) = AC/BC; Group 2 (Low‐ICM) = CA/CB. Adjusters include maternal age, BMI, infertility duration, number of treatment cycles, AFC, AMH, infertility diagnosis, the type of single blastocyst transfer cycles, EM, blastocyst developmental stage and degree of blastocyst expansion. Denominators are 2943 patients with early miscarriage. GS means gestational sac, Small GS is defined as ≤ 26 mm 6 weeks after embryo transfer. CRL means crown‐rump length, Short CRL is defined as having a germ/crown‐rump length ≤ 14 mm 6 weeks after embryo transfer. EHR means embryonic heart rate. Low EHR is defined as ≤ 120 bpm 6 weeks after embryo transfer. YS means yolk sac, Large or small YS is defined as > 6 mm or < 3 mm 6 weeks after embryo transfer.

## 4 Discussion

### 4.1 Principal Findings

The incidence of having no transferable high‐quality blastocysts was 33.8%. Maternal age, BMI, infertility duration, cycle number, embryo developmental stage, and transfer strategies are independent risk factors that affect the quality of blastocysts. Through two sensitivity analyses, our findings consistently demonstrated that group 1 exhibited a significantly higher pregnancy success rate compared to group 2, though both were significantly lower than group 0. Furthermore, comparisons of BPR, EMR, and LMR revealed that differences in pregnancy outcomes among blastocysts with varying morphological scores primarily arise during the early implantation stage. Low TE grades mainly affect the size of the gestational sac and the formation of empty gestational sacs, while low ICM grades mainly affect the size of the yolk sac. Additionally, our analysis indicated that embryo morphologic parameters are associated with the sex ratio.

### 4.2 Results in the Context of What Is Known

While one study concluded that blastocyst morphology is unrelated to miscarriage rates [24], our analysis further categorized miscarriages into biochemical pregnancies, early miscarriages, and late miscarriages. We found that biochemical pregnancy and early miscarriage rates were significantly higher in low‐grade blastocysts compared to group 0, with ICM low‐grade blastocysts exhibiting higher rates than TE low‐grade blastocysts. This finding underscores the critical role of molecular recognition and communication between blastocysts and the endometrium during early implantation [25]. Our findings are further corroborated by evidence that high‐quality blastocysts promote endometrial stromal cell migration and implantation through the secretion of specific microRNAs (miRNAs), whereas miRNAs from low‐quality blastocysts lack this effect [26].

Although the inner cell mass score has historically been considered the primary criterion for embryo selection due to its strong correlation with pregnancy outcomes [27, 28], recent studies have highlighted the predictive value of trophectoderm scores [10, 29]. Consequently, the relative importance of these scores remains debated, particularly in clinical settings where the choice between BC‐scored and CB‐scored embryos must be made in the absence of group 0. These inconsistencies may stem from variations in study methodologies, population characteristics, cycle types, and sample sizes. With the largest sample size to date, our study adjusted for 11 potential confounders, including developmental days (Day 5 and 6), blastocyst expansion grades (Grade 3–6), and transfer cycle types (fresh, biopsied, thawed, and blastocysts derived from thawed cleavage‐stage embryos). Through repeated validation using multiple statistical methods, we consistently concluded that group 1 yield significantly better pregnancy outcomes compared to group 2. At the molecular level, our findings are further supported by evidence that epiblasts, derived from inner cell mass differentiation, regulate trophectoderm maturation through TGF‐β and FGF signaling pathways during early embryo implantation [30].

Our results indicate an association between trophectoderm score and fetal sex, with low trophectoderm grade embryos exhibiting a lower male fetus rate compared to those with high trophectoderm scores, consistent with previous findings [3, 31]. This phenomenon may arise because female embryos require more glucose to inactivate the second X chromosome, leading to slower mitotic rates [32].

### 4.3 Clinical Implications

In this study, we identified several maternal and embryonic factors that significantly influence blastocyst quality, particularly in relation to TE and ICM grading. Advanced maternal age, higher BMI, longer duration of infertility, and increased number of treatment cycles were all independently associated with a higher likelihood of group 1. These findings are consistent with previous studies demonstrating that with increasing maternal age, oocyte mitochondrial function declines and the incidence of chromosomal aneuploidy rises, thereby compromising the developmental potential of early embryos [33]. Meanwhile, obesity‐related metabolic disturbances may indirectly affect embryonic development by impairing oocyte quality [34]. Prolonged infertility and repeated treatments may reflect underlying reproductive pathologies or cumulative effects of ovarian stimulation, both of which may compromise the developmental potential of embryos. Interestingly, prolonged embryonic development time was strongly associated with poor TE and ICM morphology. This may reflect delayed embryonic competence or suboptimal in vitro conditions for extended culture, which can impair blastocyst expansion, increase oxidative stress, and alter gene expression patterns critical for TE differentiation [35]. The particularly strong association between prolonged culture and low ICM grade (OR = 8.47) suggests that blastocysts requiring more time to reach full expansion may have impaired pluripotent cell mass development, possibly due to intrinsic cellular defects or asynchronous cleavage patterns.

On the other hand, higher antral follicle count (AFC) and greater blastocyst expansion degree were protective factors against low TE grade. AFC is a well‐established marker of ovarian reserve and is positively correlated with oocyte yield and embryo quality [36]. A greater degree of expansion may reflect enhanced TE fluid transport capacity and blastocoel formation, which are indicative of better TE function and cellular integrity. Cycle type also played a significant role. Compared to fresh embryo transfers, PGT cycles were associated with significantly lower rates of low TE and ICM grade blastocysts. This may be attributed to PGT cycles typically involving selection of morphologically superior blastocysts for biopsy. Finally, higher AMH levels were found to be protective against group 2. As a marker of ovarian reserve, AMH reflects the quantity and potentially the quality of the follicular pool. Women with higher AMH levels tend to produce more oocytes, increasing the likelihood of obtaining developmentally competent embryos with well‐formed ICMs [37].

In summary, the findings of this study highlight that different components of the blastocyst, namely the TE and ICM, are regulated by distinct clinical factors during development. This suggests that in clinical assisted reproductive practices, comprehensive consideration should be given to maternal age, ovarian reserve, BMI, infertility duration, cycle number, embryo developmental stage, and transfer strategies. By optimizing ovarian stimulation protocols and embryo culture conditions, blastocyst quality can be improved at its source.

### 4.4 Research Implications

Our findings demonstrate that blastocyst quality, particularly the grading of the trophectoderm and inner cell mass, is significantly associated with early gestational sac development and yolk sac morphology. Specifically, group 1 exhibited significantly higher rates of empty gestational sacs and small gestational sacs compared to group 0. This suggests that TE morphology may be a critical determinant of early placental development. The trophectoderm gives rise to the placenta and plays a pivotal role in mediating embryo‐endometrial interactions during implantation [38]. Low TE grade may reflect compromised cellular proliferation, reduced invasive capacity, or altered expression of adhesion molecules and cytokines, all of which could impair trophoblast invasion and placental formation, ultimately leading to suboptimal gestational sac development or pregnancy loss. In addition, the empty blastocyst rate of TE low‐grade blastocysts was significantly increased, which may be due to ICM differentiating more cells to generate TE to make up for the lack of TE, participating in endometrial recognition and the formation of gestational sac in the implantation stage, resulting in the restricted development of embryo ontology and the formation of empty gestational sac. The fact that human ICM is more plastic and can generate TE, which was confirmed in the in vitro differentiation [39, 40]. Live‐cell tracking revealed that epiblast cells in the human blastocyst are also able to produce trophectoderm. However, this is only a hypothesis and further mechanism verification is needed.

In addition, we observed that blastocysts with low ICM grade were associated with a significantly higher incidence of small yolk sacs compared to both group 0 and those with low TE grade. The ICM gives rise to the embryo proper and contributes to the formation of extraembryonic structures, including the yolk sac [38]. A compromised ICM may indicate reduced pluripotency, impaired cellular differentiation, or delayed embryonic development, which could manifest as abnormal yolk sac morphology. The yolk sac plays a crucial role in early embryonic nutrition and hematopoiesis^43^; thus, its abnormal development may reflect underlying embryonic insufficiency and could be an early marker of poor pregnancy prognosis.

Collectively, these findings underscore the differential contributions of TE and ICM quality to early embryonic development. While low TE grade appears to predominantly affect early placental development (reflected by abnormal gestational sac formation), low ICM grade may be more closely linked to intrinsic embryonic defects (reflected by abnormal yolk sac development). These results highlight the importance of comprehensive blastocyst grading in clinical practice, not only for predicting implantation potential but also for anticipating early pregnancy complications. Further studies integrating time‐lapse imaging, transcriptomic profiling, and clinical outcomes are warranted to elucidate the molecular mechanisms underlying these associations and to refine embryo selection strategies in assisted reproductive technologies.

### 4.5 Strengths and Limitations

A key strength of this study is its large sample size, which enabled robust statistical analysis across three groups stratified by blastocyst morphological scores. By adjusting for 11 potential confounders including developmental days (Day 5 and 6), blastocyst expansion grades (Grade 3–6), and transfer cycle types (fresh, biopsied, thawed, and blastocysts derived from thawed cleavage‐stage embryos) to further ensure data reliability. Second, we employed five propensity score weighting methods to balance baseline characteristics among the three study groups. Additionally, we eliminated extreme propensity score values and conducted sensitivity analyses on the trimmed data to enhance the robustness and accuracy of our findings. Third, we categorized pregnancy outcomes into biochemical pregnancy, clinical pregnancy, early miscarriage, late miscarriage, and live birth to precisely identify the stages at which poor reproductive outcomes occur following embryo transfer. Moreover, few studies have explored the association between blastocyst morphological parameters and intrauterine development during early pregnancy.

A limitation of this study is its retrospective design, which may introduce inherent bias. Although embryo grading was performed by 2–3 uniformly trained and experienced embryologists, we acknowledge the potential for inter‐observer variability. Additionally, group 2 had a relatively small sample size compared to the other groups, potentially introducing bias, and there are limitations in the ability to detect clinically meaningful differences. Finally, as the data were derived from a single center, future multi‐center studies with larger sample sizes are warranted to validate these findings.

## 5 Conclusion

In summary, this study systematically evaluated the clinical factors affecting blastocyst quality and their impact on pregnancy outcomes. Approximately one‐third of cycles failed to yield high‐quality blastocysts, highlighting blastocyst quality as a critical limiting factor in assisted reproductive success. Maternal age, AFC, BMI, duration of infertility, number of treatment cycles, embryonic developmental stage, and transfer strategy were identified as independent risk factors influencing blastocyst formation and quality, warranting careful consideration and intervention in clinical practice. Further analysis revealed that decreased blastocyst quality was significantly associated with lower clinical pregnancy and live birth rates, as well as higher rates of biochemical pregnancy and early miscarriage, underscoring its essential role in early pregnancy stability. Notably, different components of the blastocyst exerted distinct effects on early embryonic development: blastocysts with lower TE grades were more likely to result in empty or small gestational sacs, while those with low ICM grades were closely associated with the occurrence of small yolk sacs. These findings suggest that morphological grading of blastocysts not only predicts pregnancy outcomes but may also reflect specific developmental deficiencies. Future studies should integrate molecular biomarkers with morphological assessment to more precisely evaluate blastocyst quality and improve outcomes through optimized ovarian stimulation protocols, laboratory culture conditions, and individualized transfer strategies.

## Funding

This work was supported by the Key Research and Development Program of Hunan Province (2023SK2069).

## Disclosure

This study was a retrospective observational study using clinical records and approved by the Institutional Review Board of Reproductive and Genetic Hospital of CITIC‐Xiangya (LL‐SC‐2020‐019).

## Conflicts of Interest

The authors declare no conflicts of interest.

## Supporting information


**Figure S1:** Forest plot for comparison of continuous variable outcomes between the three groups.

**Figure S2:** Forest plot of dichotomous outcome comparisons between three groups of trimmed data.

**Figure S3:** Forest plot of continuous variable outcome comparisons between three groups of trimmed data.

**Figure S4:** Love plots.

**Figure S5:** PS overlap plots.

**Table S1:** Demographic and clinical characteristics among unweighted and five propensity score weighted patients.

**Table S2:** Comparison of pregnancy outcomes and neonatal outcomes between the three groups.

**Table S3:** Comparison of pregnancy outcomes and neonatal outcomes between the three groups of trimmed data.

**Table S4:** The counts of only‐low TE grade, only‐low ICM grade, and both‐available at the index transfer.

**Table S5:** Sensitivity analyses in the both‐available subset.

**Table S6:** Propensity score–weighted analyses of the two groups (low TE grade vs. low ICM grade).

**Table S7:** ESS.

**Table S8:** Maximum weights.

**Table S9:** Sensitivity analyses in three groups included calendar year in the confounding factors but not the culture days.

**Table S10:** Comparison of pregnancy outcomes and neonatal outcomes between the three groups within PGT cycles only.

**Table S11:** Comparison of pregnancy outcomes and neonatal outcomes between the three groups excluding all PGT cycles.

**Table S12:** Comparison of pregnancy outcomes and neonatal outcomes between the PGT and non‐PGT cycles in low TE grade group.

**Table S13:** Comparison of pregnancy outcomes and neonatal outcomes between the PGT and non‐PGT cycles in low ICM grade group.

## Data Availability

The data that support the findings of this study are available on request from the corresponding author. The data are not publicly available due to privacy or ethical restrictions.
