# Supplementary figures and images for: Incidence, Clinical Risk Factors, and Pregnancy Outcomes of Trophectoderm‐ and Inner Cell Mass–Poor‐Quality Blastocysts in Single Blastocyst Transfer Cycles: A Retrospective Cohort Study
Source: Reprod Med Biol. 2025 Dec 30;25(1):e70006. doi: 10.1002/rmb2.70006 (PMC12754268; doi:10.1002/rmb2.70006)

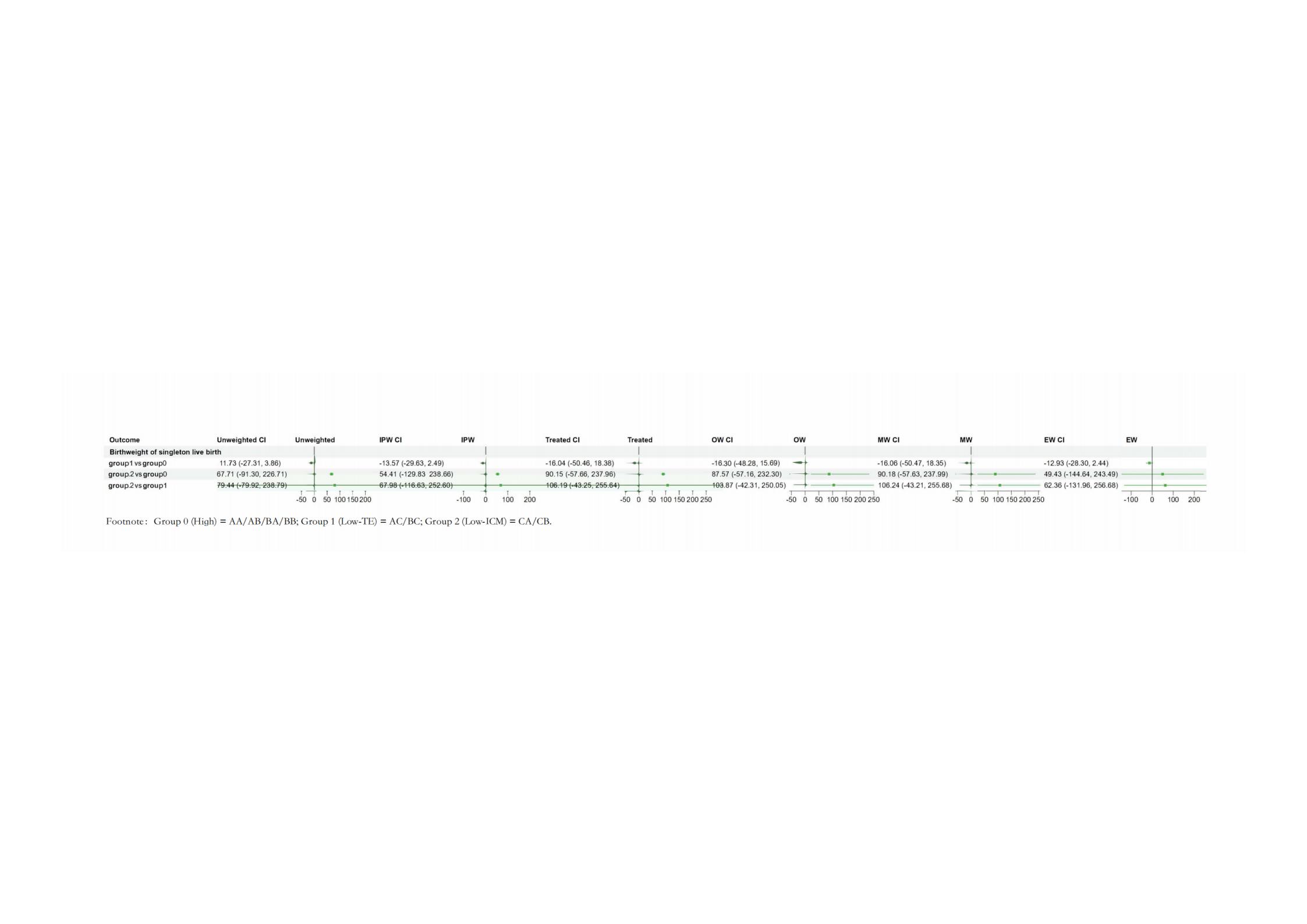

Supplement: Supplementary file 1 — Figure S1: Forest plot for comparison of continuous variable outcomes between the three groups. [file RMB2-25-e70006-s013.jpg]

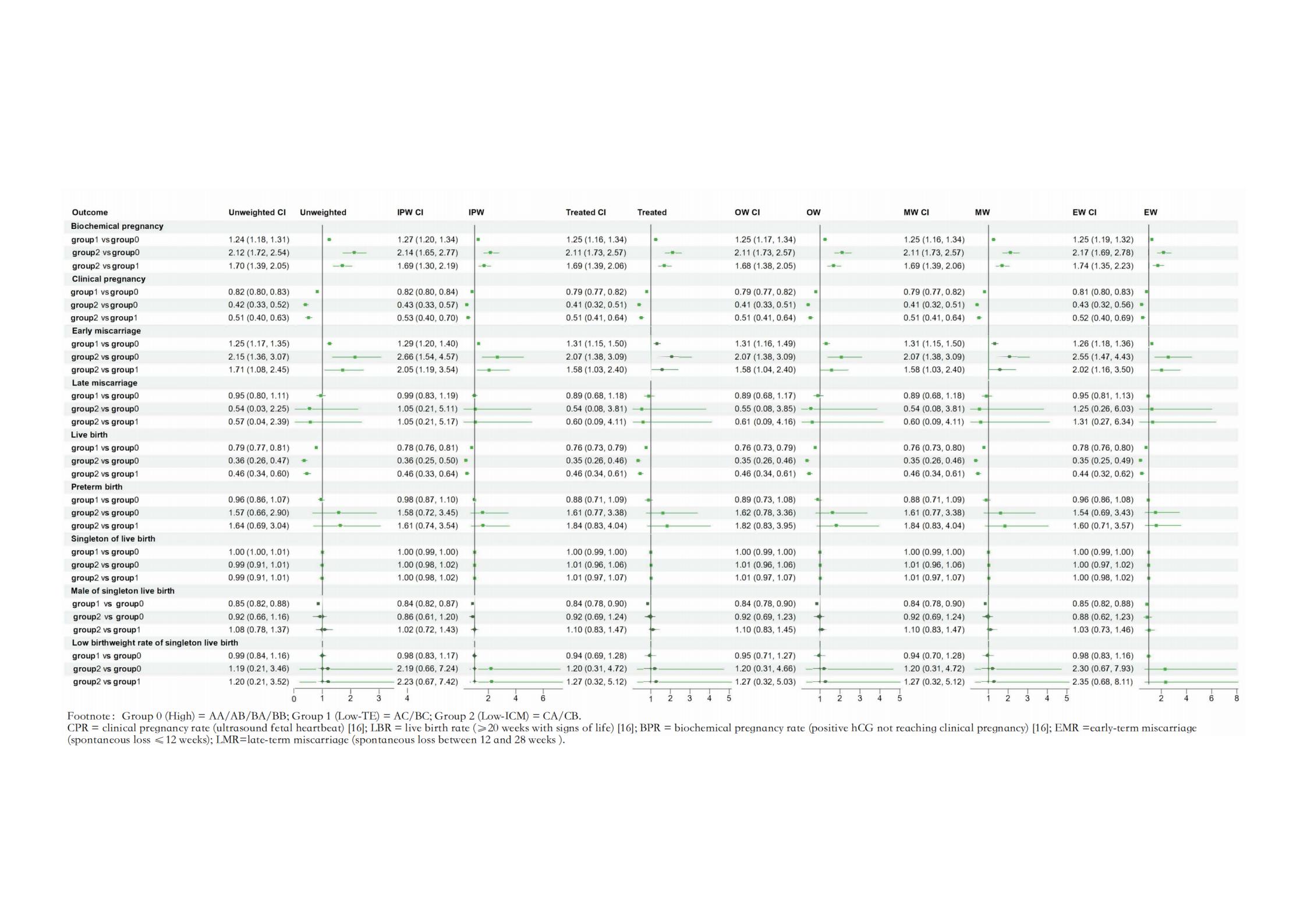

Supplement: Supplementary file 2 — Figure S2: Forest plot of dichotomous outcome comparisons between three groups of trimmed data. [file RMB2-25-e70006-s018.jpg]

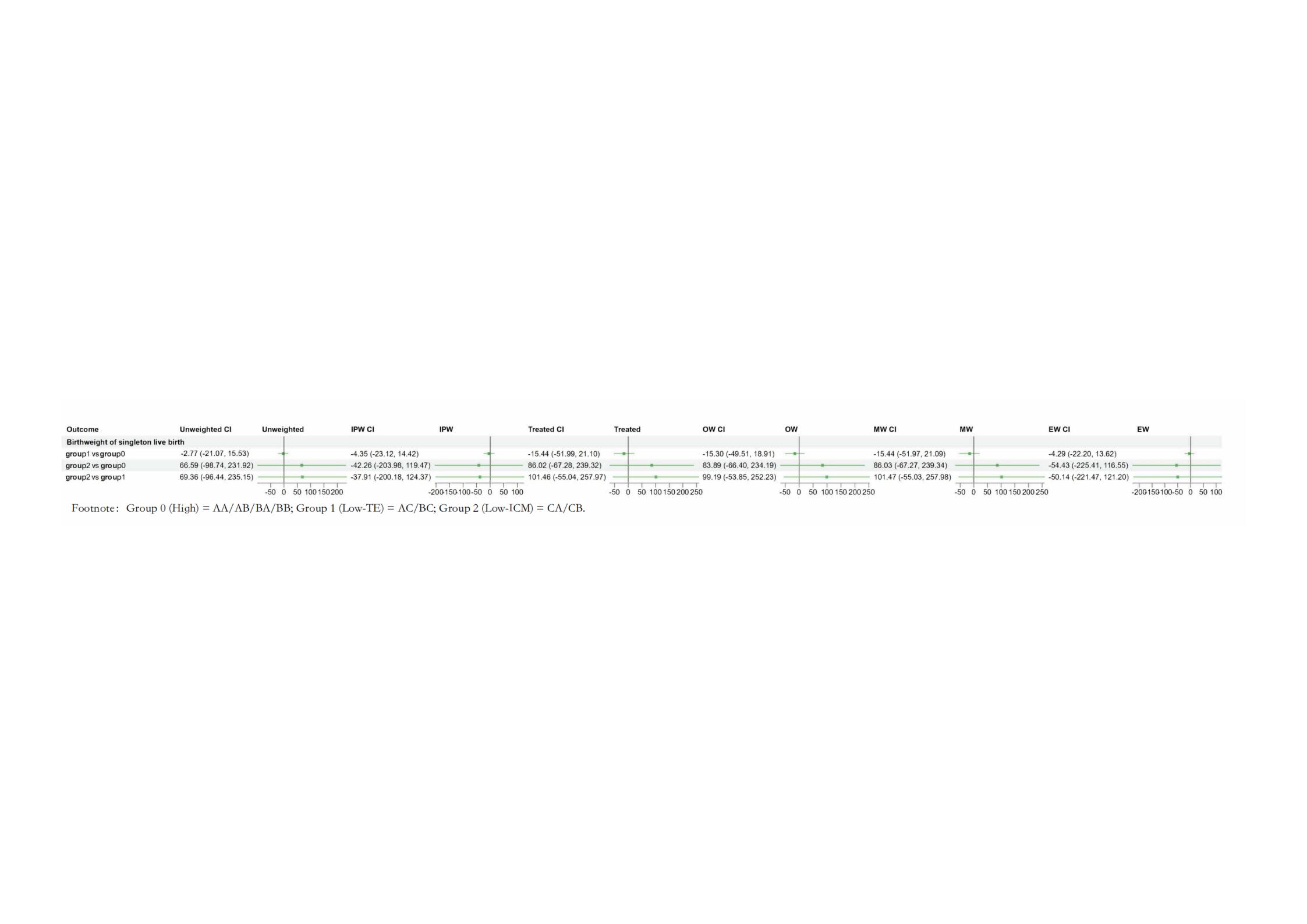

Supplement: Supplementary file 3 — Figure S3: Forest plot of continuous variable outcome comparisons between three groups of trimmed data. [file RMB2-25-e70006-s008.jpg]

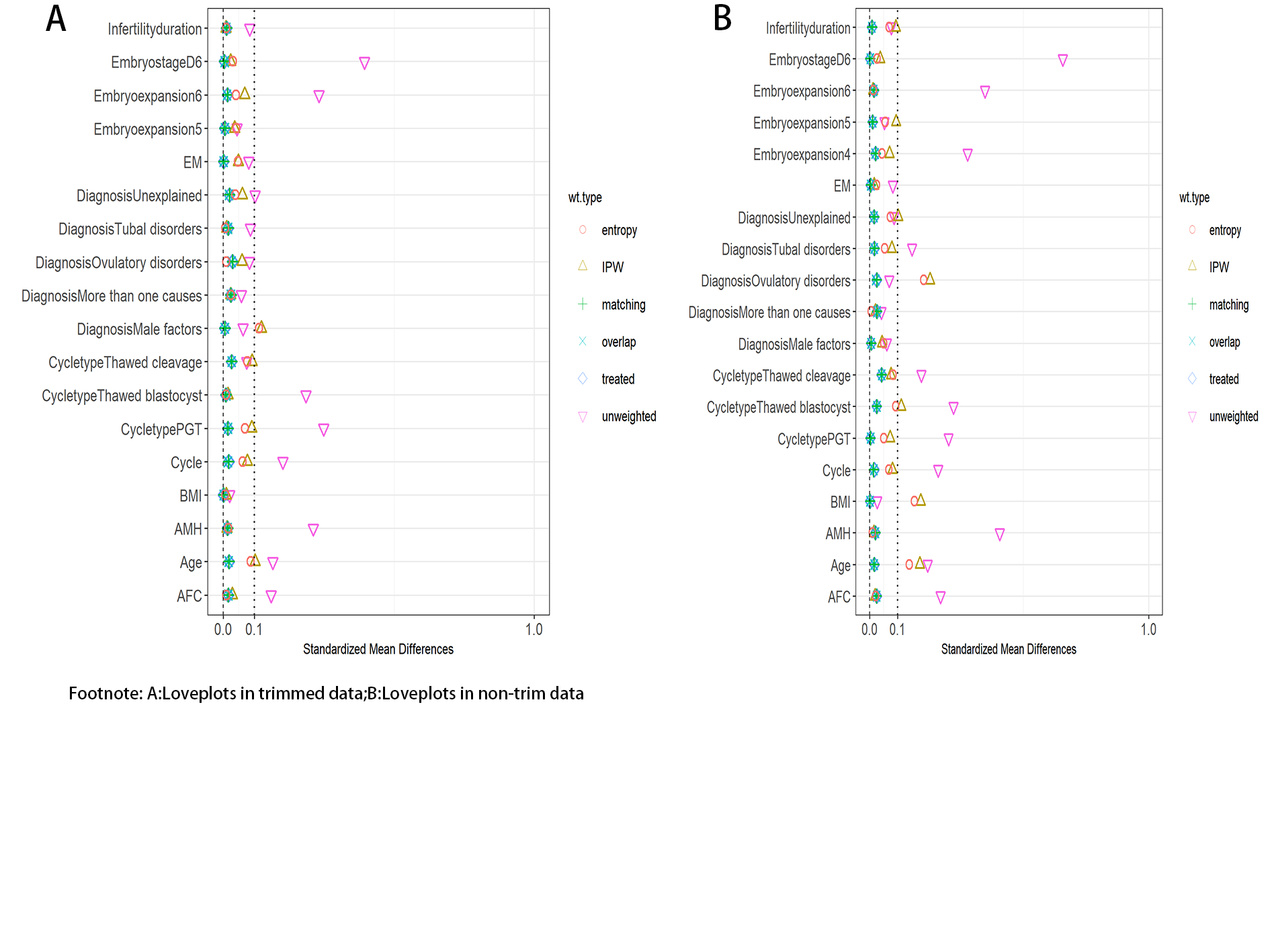

Supplement: Supplementary file 4 — Figure S4: Love plots. [file RMB2-25-e70006-s009.tif]

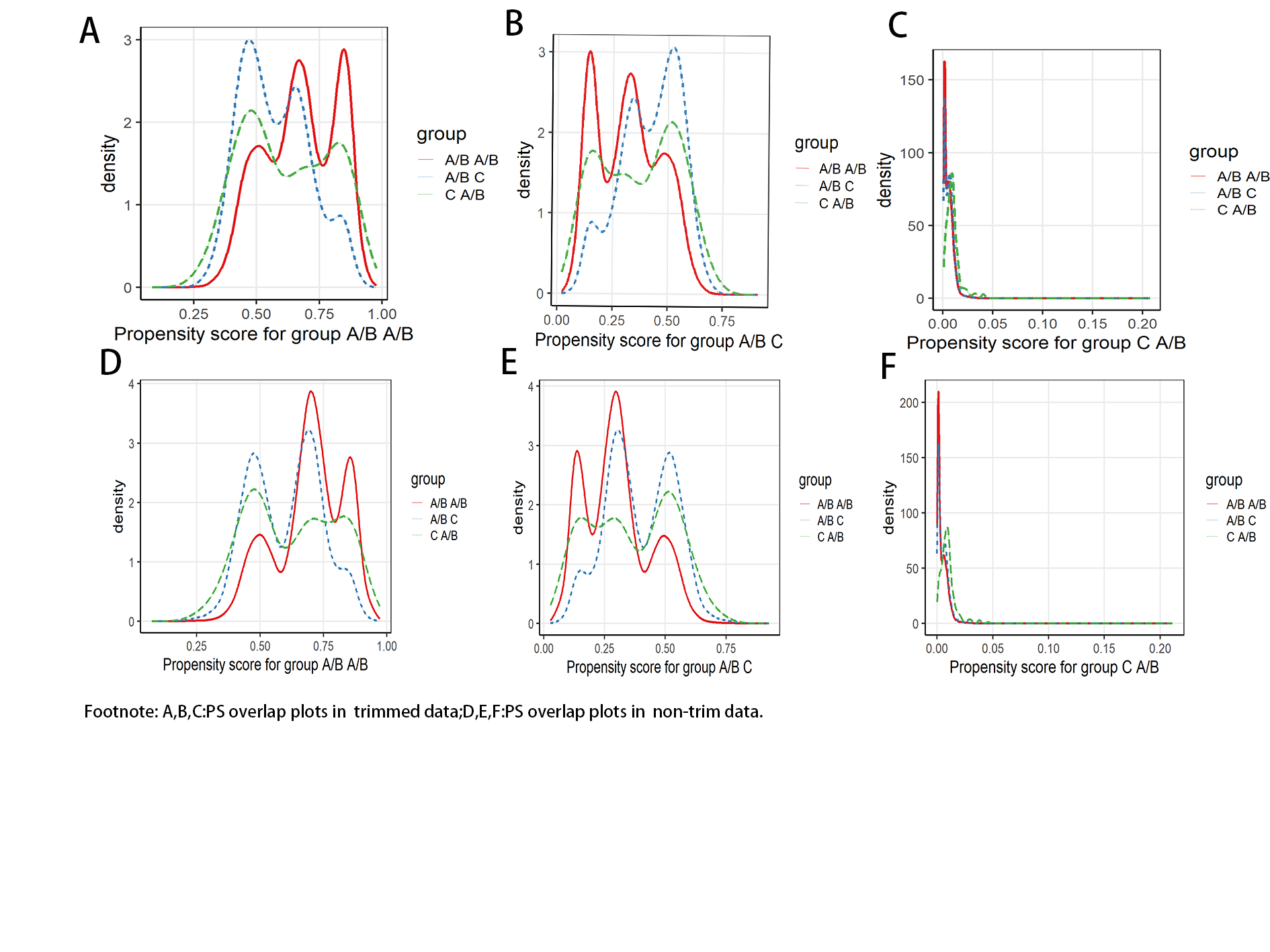

Supplement: Supplementary file 5 — Figure S5: PS overlap plots. [file RMB2-25-e70006-s015.tif]
